# Subungual Melanoma of the Thumb

**Published:** 2013-02-11

**Authors:** Nadia F. Nocera, Michael I. Baruch, Minbae Kim

**Affiliations:** ^a^New York Medical College, Valhalla; ^b^Departments of Plastic Surgery, St Joseph's Hospital, Paterson, NJ; ^c^Departments of Pathology, St Joseph's Hospital, Paterson, NJ

**Figure:**
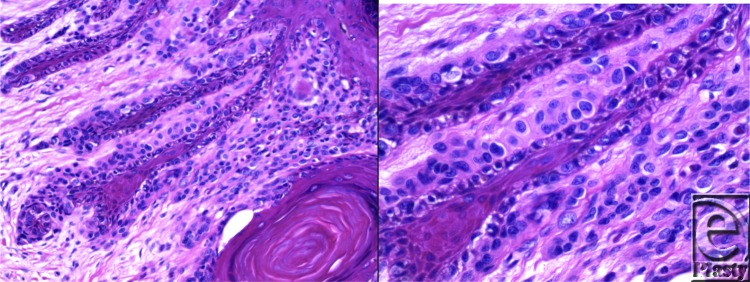


## DESCRIPTION

A 57-year-old Polish woman presented with longitudinal melanonychia of the right thumb that has been present for 1 year. The lesion has not changed in size during that period. The patient reported no history of trauma to the thumb. There was no tenderness to palpation of the area and there were no signs of infection. The patient had full range of motion of the interphalangeal (IP) joint.

## QUESTIONS

1. **What is the diagnosis?**
2. **How is the diagnosis made?**
3. **What is the treatment?**
4. **What are the options for treatment?**

## DISCUSSION

For this patient, 4-mm punch biopsy of the pigmented area was done and results were consistent with malignant melanoma in-situ. The distal phalanx of the right thumb was amputated. Subungual melanoma accounts for approximately 0.7% to 3.5% of all cases of melanoma.^1^ Signs and symptoms include nail discoloration, a nonhealing wound, a mass, a split in the nail, and bleeding.^2^ Another sign may include Hutchinson's sign, which is periungual extension of the dark pigmentation onto the nailfolds. The most frequently involved digits are the thumb and the hallux.^3^ It may resemble a fungal infection and is misdiagnosed as such. Other common misdiagnoses include subungual hematoma, pyogenic granuloma, paronychia, junctional nevus, and vascular tumor.^1^

Biopsy of a lesion suspicious for subungual melanoma is performed as a longitudinal full-thickness excisional procedure.^4^ The nail plate must be elevated from the underlying matrix. For lesions involving the lateral nail, the entire visible band must be excised in a longitudinal full-thickness specimen, including the germinal matrix in line with the lesion.^4^ If Hutchinson sign is present, excision must include the affected periungual skin in elliptical fashion.^4^ For lesions in the middle of the nail plate, a longitudinal excision of the visible band including the germinal matrix in line with the lesion must be performed.^4^

Wide local excision for subungual melanoma usually requires amputation due to the paucity of soft tissue between the tumor and the presence of bone beneath the nail.^2^ Traditionally, proximal amputations have been the favored treatment, but recent experiences have shown that there is no significant difference in the rate of survival and recurrence rate with proximal IP amputation versus metacarpophalangeal or transmetacarpal amputation.^5^ Amputation in the thumb results in a 40% reduction in hand function if carried out at the metacarpophalangeal joint and about 10% if carried out at the IP joint level.^3^ Distal amputation is sufficient if appropriate cutaneous margins can be obtained and is associated with a low risk of recurrence and no diminution in survival.^2^
